# Discovery of peptide inhibitors targeting human programmed death 1 (PD-1) receptor

**DOI:** 10.18632/oncotarget.11274

**Published:** 2016-08-12

**Authors:** Qiao Li, Lina Quan, Jiankun Lyu, Zenghui He, Xia Wang, Jiajia Meng, Zhenjiang Zhao, Lili Zhu, Xiaofeng Liu, Honglin Li

**Affiliations:** ^1^ State Key Laboratory of Bioreactor Engineering, Shanghai Key Laboratory of New Drug Design, School of Pharmacy, East China University of Science and Technology, Shanghai, 200237, China

**Keywords:** immunotherapy, human programmed death 1, peptide inhibitor, protein-protein interactions (PPIs), de novo peptide design

## Abstract

Blocking the interaction of human programmed death 1 (hPD-1) and its ligand hPD-L1 has been a promising immunotherapy in cancer treatment. In this paper, using a computational *de novo* peptide design method, we designed several hPD-1 binding peptides. The most potent peptide Ar5Y_4 showed a KD value of 1.38 ± 0.39 μM, comparable to the binding affinity of the cognate hPD-L1. A Surface Plasmon Resonance (SPR) competitive binding assay result indicated that Ar5Y_4 could inhibit the interaction of hPD-1/hPD-L1. Moreover, Ar5Y_4 could restore the function of Jurkat T cells which had been suppressed by stimulated HCT116 cells. Peptides described in this paper provide promising biologic candidates for cancer immunotherapy or diagnostics.

## INTRODUCTION

Activation of the immune system for cancer therapy has long been a goal in immunology and oncology [1, 2], and a recent breakthrough in cancer immunotherapy is the immunologic checkpoint blockade [3, 4]. One of the checkpoint receptors that have been most actively studied in the context of clinical cancer immunotherapy is PD-1 (or CD279) [5, 6], which participates in a dominant immunosuppressive pathway. PD-1 has two cognate ligands: PD-L1 (CD274, B7-H1) and PD-L2 (CD273, B7-DC) [7, 8]. Under normal conditions, binding of PD-1 to its ligands can deliver inhibitory signals to regulate the balance between T cell activation, tolerance and immunopathology [9]. However, tumor cells often overexpress PD-1 ligands to limit T cell activity and evade antitumor immune responses [10, 11]. Therefore, blocking the pathway of PD-1 and its ligands can significantly enhance T cell functions and thus eliminate cancers [12–14].

Compared with PD-L2, PD-L1 is expressed more widely, and blockade of PD-1/PD-L1 interaction is more frequently targeted by therapeutic agents [11, 15]. Initially, modulators blocking the PD-1/PD-L1 pathway are antibodies, such as Nivolumab, Pembrolizumab, MPDL3280A and MEDI4736 [16–21]. However, antibody drugs always have some issues including the high production cost, unexpected immunogenicity and bad tissue penetration [22]. Therefore, it is interesting to develop chemical or peptide-like molecules to block the PD-1/PD-L1 interaction, which hopefully provides alternative drug candidates to overcome the drawbacks of antibody-based immunotherapies. Several peptides and small organic compounds targeting PD-L1 have been reported [23, 24]. However, there is no successful report about low-molecular weight modulators targeting PD-1. Herein we wish to develop peptide inhibitors targeting human PD-1.

**Figure 1:**
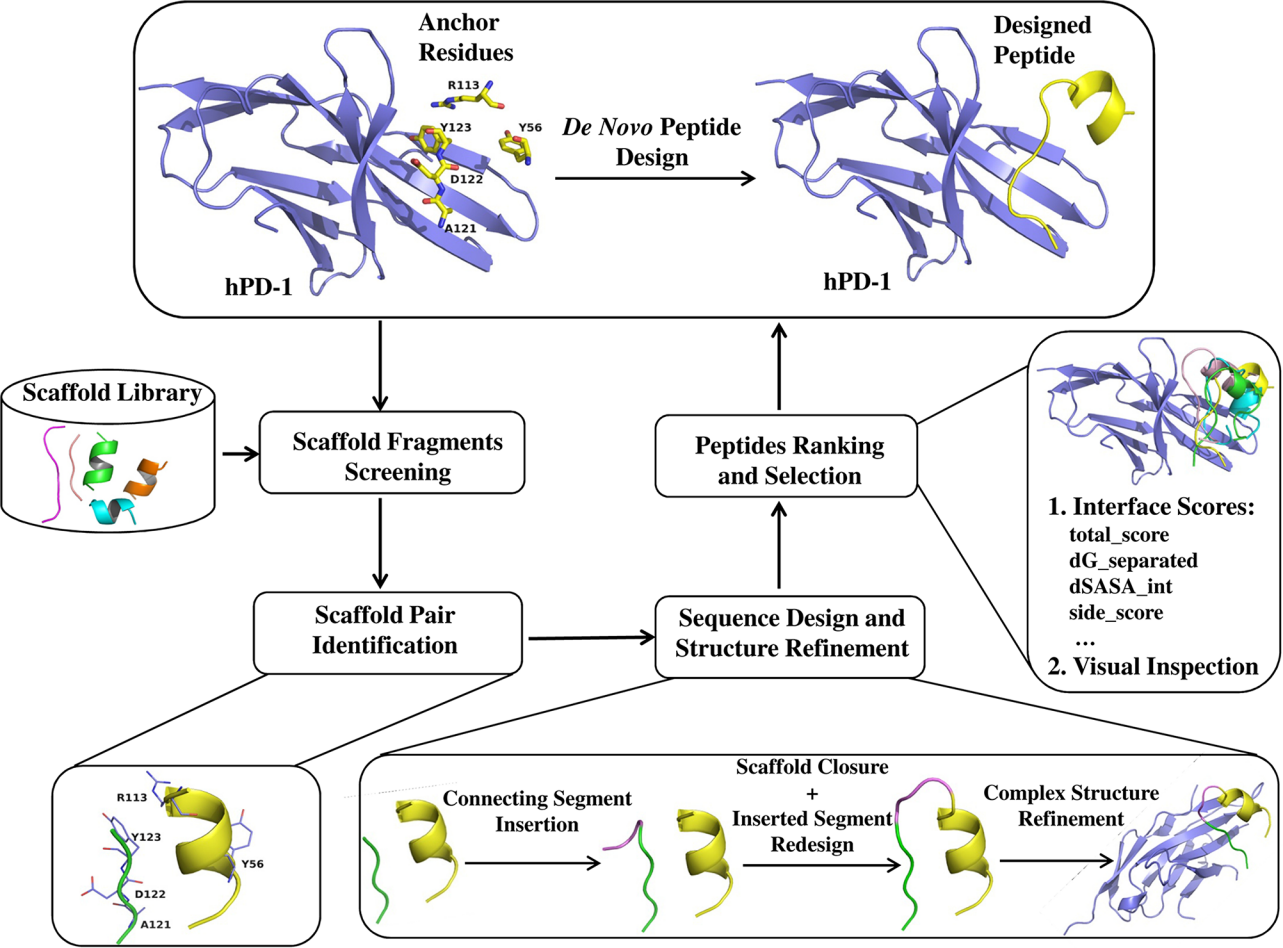
Schematic representation of workflow for *de novo* peptide design

Basing on a *de novo* peptide design method, we designed some peptide ligands of hPD-1 with the most potent peptide Ar5Y_4 showing a *K*_D_ value of 1.38 ± 0.39 μM that was comparable to the *K*_D_ value of the cognate hPD-L1. Moreover, Ar5Y_4 can effectively inhibit the binding of hPD-L1 to hPD-1 validated by a SPR competitive binding assay and restore the function of suppressed Jurkat T cells. The peptide design method is motived by the well-known theory of hotspots [25–28], which only requires a scaffold fragment library and some key anchor residues as its starting points and can be applied to design peptide ligands targeting any PPIs. The outline of the *de novo* peptide design method is illustrated in Figure 1. Peptides discovered in this paper can be utilized as the starting points for further leads optimization of hPD-1.

## RESULTS

### *De novo* design peptide ligands of hPD-1

We developed a computational method to design peptide ligands of hPD-1 with residues Y56, R113, A121, D122 and Y123 of hPD-L1 (Protein Data Bank (PDB) [29] code: 4ZQK [30]) as key anchors. These five residues have a great impact on the binding of hPD-L1 to hPD-1. Scaffold fragment library is composed of 109,805 helixes and 123,230 strand fragments, which is used for providing scaffold fragments to graft the selected key anchors. Limited by positions of the five anchors and structural features of scaffold fragments, 31 strands and 56 helices were selected from the scaffold library to bear the combination of anchors A121, D122 and Y123 and the combination of anchors Y56 and R113, respectively, which formed 513 scaffold pairs. The 513 scaffold pairs were subsequently remodeled and refined into continuous peptides, and 4 peptides were selected and chemically synthesized for further biochemical validation finally. The detail information of these 4 selected peptides is shown in Table 1.

**Table 1:** Amino acid sequence, molecular weight, purity and experimentally determined *K*_D_ value of four selected peptides

Peptide^a^	Sequence^b^	MW (g/mol)^c^	Purity (%)^d^	*K*_D_ (μM)^e^
Ar5Y_1	FN**W**DYSWKSERLKEAYDL	2350.59	96.66	3.39 ± 0.85
Ar5Y_2	FN**W**DYSLEELREKAKYK	2219.50	95.80	3.14 ± 0.92
Ar5Y_3	TE**K**DYRHGNIRMKLAYDL	2223.56	96.71	3.13 ± 0.45
Ar5Y_4	GN**W**DYNSQRAQLYNQ	1856.94	98.24	1.38 ± 0.39

a Ar5Y_1, Ar5Y_2, Ar5Y_3 and Ar5Y_4 are the four selected peptides designed with anchor residues Y56, R113, A121, D122 and Y123.

b Anchor residues are underlined, residues corresponding to anchor residue A121 are in bold.

c Calculated by mass spectrometry (MS).

d Determined by HPLC.

e *K*_D_ value is shown as the mean ± SD from three independent experiments.

### SPR-based binding studies on designed peptides and hPD-1

The SPR based assay was used to measure the binding affinities of designed peptides and hPD-1. Firstly, we checked the binding affinity of hPD-L1 to hPD- 1 aiming to confirm that the immobilized hPD-1 was functional. Our measurement showed that the binding of hPD-L1 to hPD-1 had a *K*_D_ value of 1.15 ± 0.11 μM (Supplementary Figure S2), comparable to the previous reports [23]. Therefore, the immobilized hPD-1 could be used to measure the binding affinities of designed peptides and hPD-1. The SPR binding assay results of the four designed peptides are shown in Table 1 and Supplementary Figure S3. All the *K*_D_ values of four peptides are no bigger than 5 μM and the most potent peptide Ar5Y_4 has a *K*_D_ value of 1.38 ± 0.39 μM, indicating that the *de novo* peptide design method is capable of designing peptide ligands of hPD-1 with detectable affinities.

### Peptide Ar5Y_4 inhibits the binding of hPD-L1 to hPD-1

**Figure 2:**
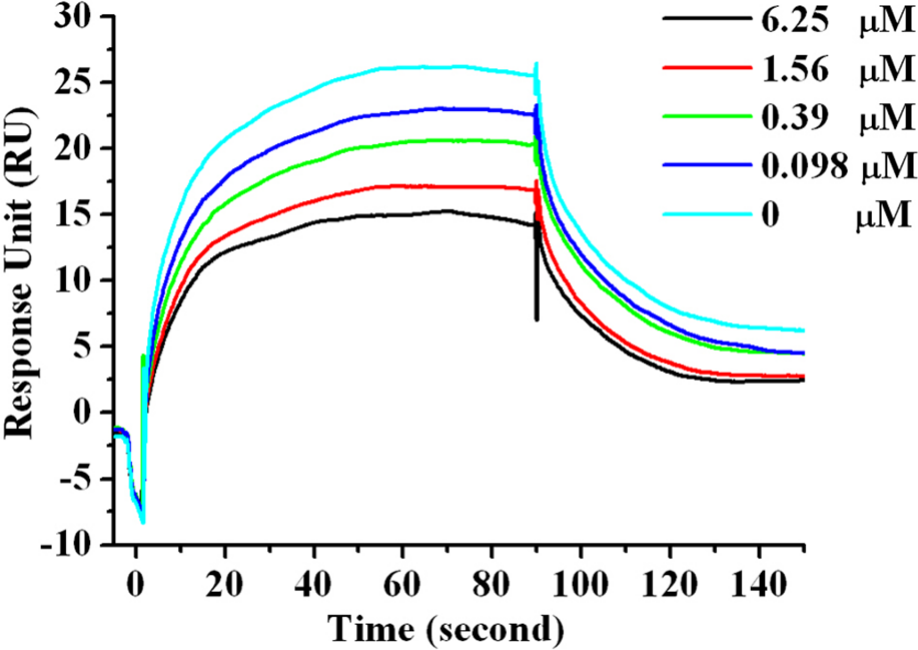
SPR competitive binding curves with increasing Ar5Y_4 concentrations (0 μM, 0.098 μM, 0.39 μM, 1.56 μM, 6.25 μM) with hPD-L1 immobilized on the sensor chip for investigating the ability of Ar5Y_4 blocking the interaction of hPD-1 and hPD-L1 Pre-incubation of Ar5Y_4 with hPD-1 effectively inhibits the binding of hPD-L1 to hPD-1.

Among the four designed peptides, peptide Ar5Y_4 has the highest binding affinity validated by the SPR direct binding assay, representing the most potent hPD-1 binding peptide. The *in vitro* activity of Ar5Y_4 was further confirmed by a SPR competitive binding assay. Pre-incubated mixtures of hPD-1 and various concentrations of Ar5Y_4 were injected over the sensor chip on which the hPD-L1 was immobilized. As shown by the RU values in Figure 2, increasing concentrations of Ar5Y_4 lead to decreasing SPR signals, indicating that Ar5Y_4 could effectively inhibit the binding of hPD-L1 to hPD- 1. Therefore, peptide Ar5Y_4 is a promising inhibitor and can be utilized as the starting point for further leads optimization.

### Effect of peptide Ar5Y_4 on IL-2 production of Jurkat T cells

**Figure 3:**
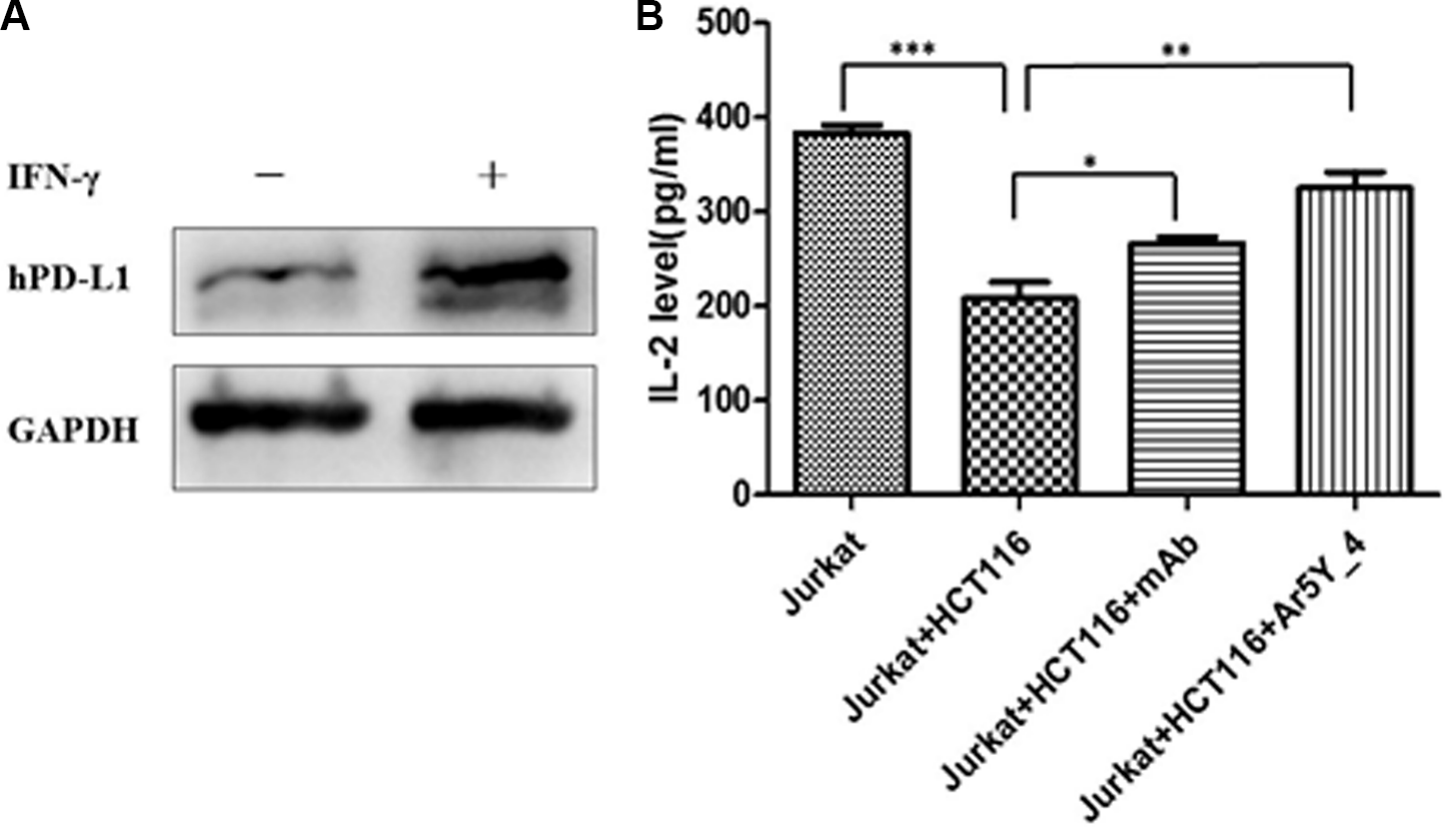
(**A**) Western blot analysis of the expression of hPD-L1 in HCT116 cells before and after being stimulated by human IFN-γ. (**B**) Effect of peptide Ar5Y_4 on IL-2 production of Jurkat T cells. The addition of IFN-γ pretreated HCT116 cells makes the Jurkat T cells production of IL-2 decrease significantly, while the addition of 250 μM peptide Ar5Y_4 could restore 67% of IL-2 production. Anti-PD-1 blocking antibody is used for reference. Results are the representative of three independent experiments. *P < 0.05; **P < 0.01; ***P < 0.001, data is analyzed using Student's *t*-test.

Cytokine production is an important indicator for T-cell function evaluation. To investigate whether peptide Ar5Y_4 can restore the suppressed function of Jurkat T cells, we assessed the T cells production of IL-2 by ELISA. Jurkat T cells can be stimulated and induce the expression of hPD- 1. Meanwhile, HCT116 cells can upregulate the expression of hPD-L1 after being stimulated by IFN-γ (Figure 3A). The activated Jurkat T cells production of IL-2 decreases significantly when Jurkat T cells are co- cultured with IFN-γ pretreated HCT116 cells (Figure 3B). HCT116 cells can suppress the function of Jurkat T cells attributing to the binding of hPD-L1 to hPD-L1. Figure 3B shows that the addition of 250 μM peptide Ar5Y_4 restores 67% of the Jurkat T cells production of IL-2. Therefore, peptide Ar5Y_4 can restore the suppressed function of Jurkat T cells by blocking the interaction of hPD-1 and hPD-L1.

## DISCUSSION

**Figure 4:**
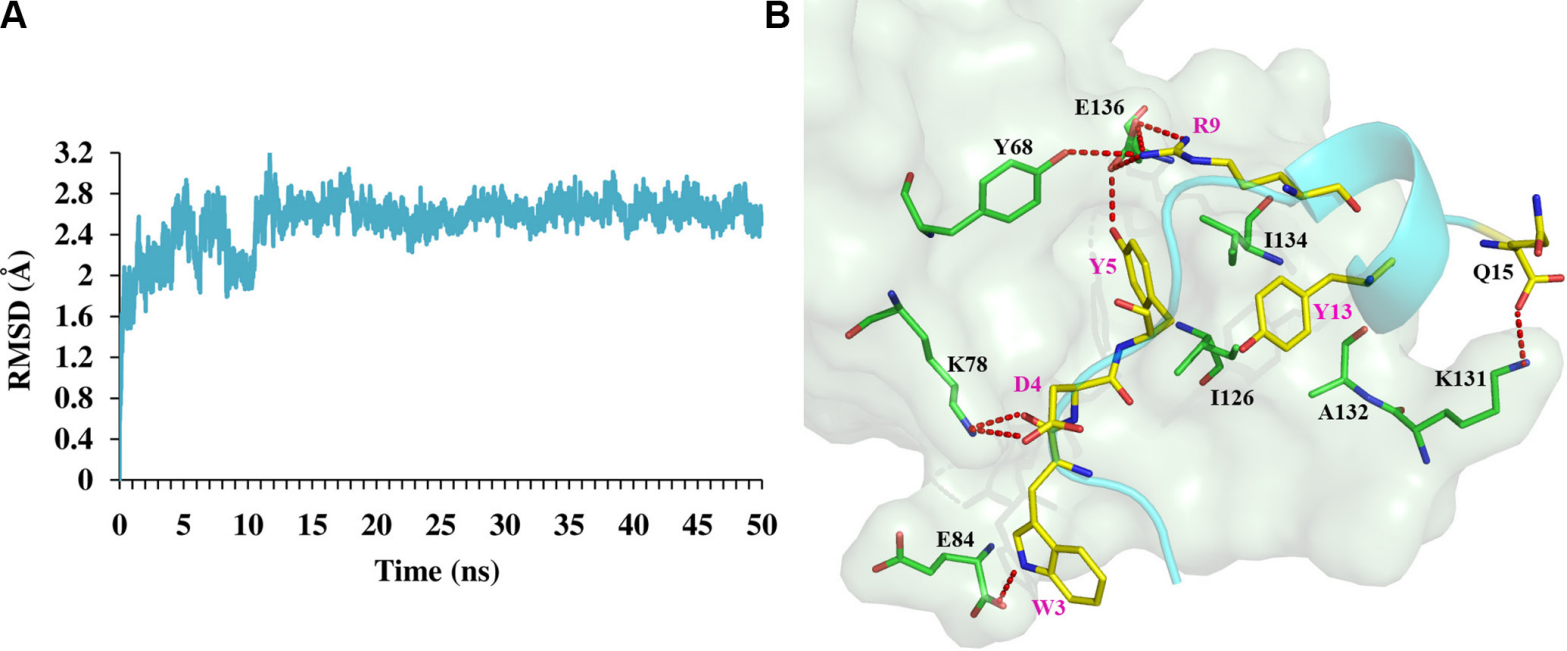
MD simulation results of peptide Ar5Y_4 in complex with hPD-1 (**A**) Time-course of RMSDs of backbone atoms against the initial designed structure of Ar5Y_4 in complex with hPD-1. (**B**) Binding model of Ar5Y_4 with hPD-1 predicted by MD simulation. hPD-1 is represented by palegreen surface and residues in hPD-1 that are important for the interaction are shown as green lines; Peptide Ar5Y_4 is shown in cyan cartoon and all residues in Ar5Y_4 important for the interaction are depicted as yellow lines. Hydrogen bonds are depicted as red dashed lines.

Peptides targeting PPIs show high binding affinity and specificity considering the interfacial features of PPIs [31, 32]. Taking advantage of a *de novo* peptide design method, we successfully designed peptide ligands of hPD- 1. All the four selected peptides show micromolar binding affinities, and the SPR competitive assay validates that the most potent peptide Ar5Y_4 could inhibit the binding of hPD-L1 to hPD-1. Furthermore, Ar5Y_4 could restore the function of suppressed Jurkat T cells. To generate the putative binding mode of Ar5Y_4, a 50 ns MD simulation was conducted with the designed model of Ar5Y_4 in complex with hPD-1 as the initial structure (Figure 4A).

In the predicted MD simulation model, almost all the key interactions between Ar5Y_4 and hPD-1 are conducted by the five anchor residues (W3, D4, Y5, R9 and Y13) in Ar5Y_4 (Figure 4B): salt bridges (D4-K78 and R9-E136), hydrogen bonds (W3-K78, W3-E84, Y5-E136, R9-Y68 and R9-E136) and some hydrophobic packing (Y5-Y68, Y5-I126, Y5-I134, Y13-A132 and Y13-I134). To investigate whether anchor residues contribute to the binding affinity of Ar5Y_4 and hPD-1 as the modeled structure suggested, experimental alanine mutations were performed by mutating these anchor residues to alanine, respectively. Table 2 shows the *K*_D_ values of Ar5Y_4 mutants measured by the SPR binding assay (Supplementary Figure S4). The five Ar5Y_4 mutants show reduced binding affinities to hPD-1, indicating that anchor residues in Ar5Y_4 do contribute significantly to the binding affinity.

**Table 2:** Molecular weight, purity and experimentally determined *K*_D_ value of the five Ar5Y_4 mutants

Mutant	MW (g/mol)^a^	Purity (%)^b^	*K*_D_ (μM)^c^
W3A	1741.81	97.56	8.08 ± 0.08
D4A	1812.93	95.18	18.94 ± 1.10
Y5A	1764.85	96.76	20.15 ± 0.98
R9A	1771.83	96.65	21.20 ± 1.56
Y13A	1765.85	98.91	10.23 ± 1.35

a Calculated by mass spectrometry (MS).

b Determined by HPLC.

c *K*_D_ value is shown as the mean ± SD from three independent experiments.

It should be noted that the RU values of our SPR assay results are not very high and the curves are not very smooth. However, it does not mean that the SPR assay results are unreliable. Recently, in 2015, Chang et al. [23] designed some D-peptide antagonists of hPD-L1, and the RU values of their SPR assay results were not very high. The low RU values and rough SPR binding curves can be attributed to the unstable structures of designed peptides. Though we used a helix fragment to construct the designed peptide, the helical region may be too short to maintain the stable secondary structure, which had been validated by a circular dichroism spectra experiment (relevant data is not shown in this article).

As stated above, using the combination of anchors Y56, R113, A121, D122 and Y123, we successfully designed hPD-1 binding peptides. But whether this combination is the optimal choice for designing peptide ligands of hPD-1? To investigate this question, we designed some additional peptides with three other combinations: (1) A121, D122 and Y123; (2) R113, A121, D122 and Y123; (3) R113, M115, A121, D122 and Y123. In each scenario, two peptides were selected for chemical synthesis and subjected to the SPR binding assay (Supplementary Figure S5). Detail information of representative peptides is described in Table 3.

**Table 3:** Peptides designed with additional anchor combinations

Peptide^a^	Sequence^b^	MW (g/mol)^c^	Purity (%)^d^	*K*_D_ (μM)^e^
Ar3_ref	**A**DYK	495.54	95.17	370.40 ± 2.92
Ar3_1	**W**DYD	597.59	98.40	22.35 ± 0.34
Ar4_1	G**I**DYEERWK	1195.31	95.14	28.28 ± 0.91
Ar4_2	**L**DYDGRLSQ	1066.14	96.46	83.90 ± 1.90
Ar5M_1	**L**DYGDKREGQMAE	1511.64	98.81	21.60 ± 1.03
Ar5M_2	**L**DYVNRRKMYQ	1485.74	96.08	3.32 ± 0.67

Amino acid sequence, molecualr weight, purity and experimentally determined *K*_D_ value are listed.

a Ar3_1 is designed with anchor residues A121, D122 and Y123, and Ar3_ref is the reference extracted from residues 121 to 124 of hPD-L1; Ar4_1 and Ar4_2 are peptides designed with anchor residues R113, A121, D122 and Y123; Ar5M_1 and Ar5M_2 are designed with anchor residues R113, M115, A121, D122 and Y123.

b Anchor residues are underlined, residues corresponding to anchor residue A121 are in bold

c Calculated by mass spectrometry (MS).

d Determined by HPLC.

e *K*_D_ value is shown as the mean ± SD from three independent experiments.

In the case of using residues A121, D122 and Y123 as anchors, the representative design is Ar3_1 with a sequence of WDYD. Ar3_ref is the reference oligo-peptide extracted from residues 121 to 124 of hPD-L1 (sequence: ADYK). Interestingly, Ar3_1 binds to hPD- 1 more tightly (*K*_D_ = 22.35 ± 0.34 μM) than Ar3_ref (*K*_D_ = 370.40 ± 2.92 μM), which can be attributed to the large aromatic side chain of tryptophan in Ar3_1 that increases the binding affinity of Ar3_1 and hPD-1 greatly. Ar4_1 and Ar4_2 are peptides designed with anchors R113, A121, D122 and Y123, from which the removal of anchor Y56 reduces the binding affinities a lot. According to the hotspot prediction results, residue M115 in hPD-L1 is also predicted as a hotspot, which we do not show in this article. Ar5M_1 and Ar5M_2 are representative peptides designed with anchor residues R113, M115, A121, D122 and Y123. Though Ar5M_1 and Ar5M_2 share same anchors, their *K*_D_ values are different greatly (21.60 ± 1.03 μM and 3.32 ± 0.67 μM, respectively). The residue behind residue M9 corresponding to anchor M115 in Ar5M_2 is a tyrosine (Y10), which can make Ar5M_2 be regarded as a peptide designed with anchor residues Y56, R113, A121, D122 and Y123. Thus, from this perspective, the combination of anchors R113, M115, A121, D122 and Y123 is inferior to that of Y56, R113, A121, D122 and Y123. Overall, comparing with three other combinations, the combination of Y56, R113, A121, D122 and Y123 is the optimal hotspots for hPD-1 binding peptides design. Anchor residues used in the peptide design method are the most important factor determining the binding affinity, providing key interaction of the designed peptide binding to receptor protein.

In summary, using the *de novo* peptide design method, we designed some hPD-1 binding peptides with five identified residues Y56, R113, A121, D122 and Y123 derived from cognate ligand hPD-L1 as anchors. The computational peptide design method which only requires a scaffold fragment library and some key anchor residues as its starting points is practical and successful in designing hPD-1 binding peptides. The most potent peptide Ar5Y_4 shows an equivalent binding affinity of hPD-L1 and could inhibit the binding of hPD-L1 to hPD-1, providing a promising starting point for further optimization of hPD-1 peptide inhibitors. Moreover, the *de novo* peptide design method described here can be generally used to design peptide ligands targeting any PPIs guided by appropriate anchor residues.

## MATERIALS AND METHODS

### Computational peptide design

The computational *de novo* peptide design method can be divided into four stages: (i) key anchor residues identification and scaffold library construction; (ii) scaffold screening to identify scaffold fragments which the selected anchor residues can be transferred onto; (iii) sequence design and structure refinement, in which some protocols of Rosetta molecular modeling package [33, 34] are used to fulfill the work of peptide design and optimization, including Kinematic loop modeling [35, 36], Backrub [37] and Relax [38]; (iv) designs ranking and selection for experimental validation.

### Scaffold library construction and anchor residues identification

The peptide scaffold library is composed of helix and strand fragments. The reason that we use helices and strands which can form sheets with other strands to construct the designed peptides is that they are stable in structural relatively (Supplementary Table S1). All the scaffold fragments were extracted from 22,912 protein crystal structures in the PDB. The detail process of scaffold library preparation can be found in Supporting Information (Supplementary Figure S1). Anchor residues can be either identified by experimental approach like alanine-scanning mutagenesis [26] or computational prediction methods (Robetta [39], KFC2 [40], PredHS [41], HotPoint [42], MM/PB(GB)SA [43], FoldX [44] and so on). In this study, the crystal structure of hPD-1/hPD-L1 complex (PDB code: 4ZQK) [30] was used as the input structure and three *in silico* hotspot prediction tools: Robetta, KFC2 and PredHS were used to determine the anchor residues that could be used for designing hPD- 1 binding peptides. According to the predicted results, interfacial residues Y56, R113, A121, D122 and Y123 in hPD-L1 were selected as key anchors (Supplementary Figure S6, Supplementary Table S2), which corresponded to the previous structural analysis [30].

### Scaffold fragments screening and identification

Each scaffold fragment in the scaffold library was superposed onto the aforementioned anchor residues to minimize the root-mean-square deviation (RMSD) between the corresponding C_α_ and C_β_ atoms of any scaffold fragment and anchor residues using the Cealign algorithm [45] in Pymol [46], and a scaffold fragment was kept only if the RMSD was smaller than 2.0 Å. Then original residues at the superposed positions of selected scaffold fragments were replaced by the corresponding anchor residues. During the process of residue mutations, we did a special treatment to anchor residue A121 considering that its side-chain is a methyl group. Original residues in scaffold fragments which were superposed onto anchor residue A121 were kept in order to introduce extra interactional types except backbone hydrogen bond at this position observed in the structure of hPD-1/hPD-L1 complex.

Potential scaffold fragment candidates that can be used to construct peptides were identified after the process of scaffold fragment alignment and anchor residue mutation. Influenced by structural features of scaffold fragments and relative positions of anchors Y56, R113, A121, D122 and Y123, a single scaffold fragment was unable to bear all the five selected anchors. Therefore, we used two scaffold fragments to bear them. For convenience, the two fragment combination is referred to as “scaffold pair” in the following part. It should be noted that not every retrieved scaffold pair can be used for the reason that they may have steric clashes with the receptor protein or each other. Therefore, it is necessary to conduct a steric clash filter to remove those steric incompatible scaffold pairs, on condition that the distance of any two heavy atoms from two different fragments is smaller than the sum of their van der Waals radii scaled by 0.75.

### Sequence design and structure refinement

For the matched scaffold pair, there was a backbone discontinuity between them. To make the scaffold pair be a continuous peptide, we used a backbone building strategy that performed oligo-peptide segment insertion and Kinematic loop modeling [35, 36] step. The newly built connecting segment was further optimized through sequence design and structure minimization with the Backrub [37] module. The detail of this implementation can be found in Supporting Information.

### Designs ranking and selection

The resulting peptides in complex with hPD-1 were refined using Rosetta's Relax [38] module and scored using InterfaceAnalyzer module of Rosetta. According to the interface sores calculated by the InterfaceAnalyzer module, better designed peptides were selected and subjected to visual inspections aiming to filter designs with no regular secondary structures or anchor residues' side-chain orientations deviating greatly from the initial defined positions. Of the remaining designed peptides, some contained hydrophobic residues in positions that were primarily solvent-exposed. In most cases, these residues were replaced by performing fixed backbone design, allowing only polar amino acids.

### Molecular dynamics (MD) simulation

The designed model of peptide Ar5Y_4 in complex with hPD-1 served as the initial structure for MD simulation using AMBER14 [47]. The complex was solvated by TIP3P explicit waters [48] and counter ions were added to the cubic boxes. The system was minimized by steepest descent method for the first 40,000 steps, and conjugate gradient algorithm for another 20,000 steps. After minimization, the system was gradually heated from 0 to 300 K in 50 ps at constant volume and equilibrated at 300 K for another 50 ps, followed by 4 ns equilibration in the NPT ensemble. Finally, a 50 ns MD simulation without any restriction was performed at constant pressure, and the coordinates of atoms were saved every 1 ps. During the simulation, the SHAKE algorithm [49] was applied to constraint all bonds involving hydrogen atoms, and a time step of 2 fs was adopted. The Langevin thermostat [50] was used to control temperature and the Particle Mesh Ewald (PME) method [51] was applied to treat the long-range electrostatic interactions. The cutoff of distances for the long-range electrostatic and van der Waals energy terms was set to 10.0 Å. Upon completion, the output trajectory was visually inspected, along with the root-mean-square deviations (RMSDs) trace of the complex. The corresponding coordinate sets of the MD trajectory from the last 1 ns were averaged and minimized as the final MD predicted complex model.

### Protein expression and purification

The DNA sequence coding the extracellular region of hPD-1 (amino acids 34–150) was cloned into the pET28a vector between NCoI and NdeI sites. Recombinant hPD-1 was expressed in *E.coli* BL21 (DE3) cells. Cells were cultured in TB medium at 37°C until an optical density at 600 nm of 0.5–0.6 was reached, and then induced with 0.5 mM isopropyl β-D-1-thiogalactopyranoside at 37°C for 5 hours. The cells were pelleted at 4000 rpm for 30 minutes, and the pellets were suspended and lysed in the lysis buffer (50 mM Tris-HCl, pH 8.0, 50 mM NaCl, 1 mM DTT, 0.5 mM EDTA, 5% glycerol, 0.5% Triton X-100). Inclusion bodies were recovered by centrifugation (12,000 rpm for 30 minutes), and washed 3 times with 20 mM Tris-HCl, pH 8.0, 2 M urea, 2.5% Triton X-100. The inclusion bodies were finally solubilized in 20 mM Tris-HCl pH 8.0, 8 M urea. The solubilized hPD-1 was refolded by rapid dilution into 50 mM Tris-HCl, pH 8.0, 500 mM L-Arg, 24 mM NaCl, 1 mM KCl, 1 mM EDTA under magnetic stirring for 24 h. The refolding mixture was then concentrated and purified by a HiTrap SP FF cation exchange column (GE Healthcare) and a Superdex 75 gel filtration column (GE Healthcare). The purity of the refolded hPD-1 was evaluated by SDS-PAGE.

### Surface plasmon resonance analysis

Peptides were synthesized via the Fmoc protected amino acid solid-phase synthesis method [52], and the purities were bigger than 95%. The binding affinities between hPD-1 and designed peptides or hPD-L1 (Sino Biological Inc. #10084-H08H-200) were assayed using a surface plasmon resonance based biosensor instrument (Biacore T200, GE Healthcare, Sweden). The purified active hPD-1 was diluted in 10 mM sodium acetate buffer (pH 4.5) to the final concentration of 50 μg/ml. The diluted hPD-1 was immobilized on a CM5 sensor chip by amino coupling reagent kit, and the immobilization level was 5000 response unit (RU). Binding experiments were performed in PBS-P buffer (8 mM Na_2_HPO_4_, 2 mM KH_2_PO_4_, 137 mM NaCl, 2.7 mM KCl, 0.005% surfactant P20, pH 7.4) at 25°C with a flow rate of 30 μl/min. To determine the binding affinities of designed peptides (or hPD-L1) and hPD-1, gradient concentrations of peptides or hPD-L1 were injected into the channel for 90 s, followed by disassociation for 120 s. RU values were collected and all the experimental data was globally analyzed by a steady-state model within Biacore T200 Evaluation software, version 2.0.

A competitive SPR binding study was performed to test whether the designed peptide could inhibit the hPD-1/hPD-L1 interaction. The purchase hPD-L1 with a concentration of 50 μg/ml was immobilized on a CM5 sensor chip, and the immobilization level was 3780 RU. 1 μm hPD-1 was incubated for 30 min with various concentrations of the designed peptide in PBS-P buffer (8 mM Na_2_HPO_4_, 2 mM KH_2_PO_4_, 137 mM NaCl, 2.7 mM KCl, 0.005% surfactant P20, pH 7.4). The mixtures with various concentrations of the designed peptide were then injected over the chip under the same condition that was used for the SPR direct binding study.

### Cell lines

Human colon carcinoma cell lines HCT116 and human Jurkat T cells were purchased from The American Type Culture Collection (ATCC) and used for *in vitro* experiments. They were cultured in RPMI-1640 medium containing 10% fetal bovine serum (FBS) and grown in 5% CO_2_ at 37°C.

### Cell stimulation

HCT116 cells were grown to about 80% confluence and stimulated with 500 U/ml recombinant human IFN-γ (Novoprotein Scientific Inc. #C014) for 48 hours. The expression of hPD-L1 in HCT116 cells was detected by Western Blot before and after being stimulated. Jurkat T cells were stimulated with 200 ng/ml PHA and 10 ng/ml PMA.

### Detection of IL-2 production in Jurkat T cells

HCT116 cells and Jurkat T cells were co-cultured in the presence or absence of additives (10 μg/ml anti-PD-1 mAb (eBioscience #16–9989) or 250 μM peptide Ar5Y_4) in complete RPMI-1640 medium for 24 hours. Jurkat T cells alone were used as the reference. Supernatants were harvested and assessed for IL-2 by ELISA (Thermo scientific #EH2IL2).

## SUPPLEMENTARY MATERIALS FIGURES AND TABLES



## Abbreviations

hPD-1: human programmed death 1
hPD-L1: human programmed death ligand 1
hPD-L2: human programmed death ligand 2
SPR: Surface Plasmon Resonance
PPIs: Protein-protein interactions
PDB: Protein Data Bank
RU: response unit
RMSD: root-mean-square deviation
MD: molecular dynamics
RMSDs: root-mean-square deviations
PME: Particle Mesh Ewald
