# Supplementary material for: Discovery of peptide inhibitors targeting human programmed death 1 (PD-1) receptor
Source: Oncotarget. 2016 Aug 12;7(40):64967–76. doi: 10.18632/oncotarget.11274 (PMC5323130; doi:10.18632/oncotarget.11274)
Supplement: Supplementary file 1 [file oncotarget-07-64967-s001.pdf]

Discovery of peptide inhibitors targeting human programmed death 1 (PD-1) receptor

Supplementary Materials

SUPPLEMENTARY METHOD OF COMPUTATIONAL PEPTIDE DESIGN

Scaffold library preparation

All the scaffold fragments in the scaffold library were extracted from 22,912 protein crystal structures in the PDB on the basis of several filter criteria: (1) only structures solved by X-ray diffraction with overall resolution better than 2.5 Å and *R* factor smaller than 0.25 were considered; (2) non-protein chains (ligands or nucleotides) were eliminated; (3) protein structures were clustered with a 90% sequence identity, and only the centroid structures were selected. Stride [1] was used to assign secondary structure types for the selected protein structures. Peptide fragments with three or more consecutive residues assigned as "E" by Stride were treated as strands, while fragments with at least four consecutive residues assigned as "H" were treated as helices. Any scaffold fragment longer than 30 residues or containing non-standard amino acids was discarded. To further reduce the sequence redundancy of scaffold fragments, the BLAST program [2] was used to remove similar scaffold fragments with a 50% sequence identity cutoff. Figure S1 shows the length distribution of scaffold fragments in the scaffold library.

Sequence design and structure refinement

This stage can be divided into four steps: connecting segment insertion, scaffold pair closure, connecting segment redesign and complex structure refinement. In this stage, only the sequence of inserted connecting segment is redesigned, whereas the sequence of linked scaffold fragment is kept fixed.

Connecting segment insertion

For the matched scaffold pair, there was a backbone discontinuity between them. To make the scaffold pair be a continuous peptide, an oligo-peptide segment was inserted into the discontinuous position. The amino acid number of connecting segment was determined by investigating the length range between α-carbons of different residue numbers. Considering the structural features of α-helix and β-strand fragments, we only collected statistics coming from α-helix and β-strand fragments of 3873 protein crystal structures with resolution < 2.0 Å, *R* factor < 0.2 and sequence identity < 20%. By calculating the average value of length data coming from α-helices and β-strands, respectively, we determined the length range between α-carbons of different residue numbers (Table S1).

It is preferable that the inserted connecting segment is a β-strand or α-helix fragment, for that α-helices and β-strands could make the designed peptide more stable in structural comparing with the irregular loop segment. The secondary structure type of the connecting segment depends on the secondary structure type of the scaffold fragment to which a connecting segment will be appended. For example, if the connecting segment will be appended to the C-terminal of a helical scaffold fragment, the secondary structure type of the connecting segment will preferably be helical.

Scaffold pair closure

The Kinematic Loop Modeling module [3, 4] in Rosetta [5, 6] was used to reconstruct and refine the connecting segments aiming to close the gaps between scaffold pairs with the inserted connecting segments. The command line of the Kinematic Loop Modeling was:

loopmodel.linuxgccrelease -database $PATH_TO_DB -in:file:s start.pdb -loops:remodel perturb_kic -loops:refine refine_kic -loops:loop_file loop_file -in:file:fullatom -out:file:fullatom -out:overwrite -ex1 -ex2 -out:nstruct 5 -out:pdb_gz

Connecting segment redesign

The Backrub application [7] in Rosetta was used for sequence design and structure refinement of the connecting segments after loop remodeling. The residue in the connecting segment was allowed to be mutated to any amino acid type except cysteine. The command line of the Backrub was:

backrub.linuxgccrelease –database $PATH_TO_DB –s start.pdb –resfile resfile_file -ignore_unrecognized_res -pivot_residues pivot -nstruct 5

Complex structure refinement

After the process of connecting segment redesign, a continuous peptide was obtained. However, some steric clashes may exist in the interfaces of the designed peptides

and receptor protein. The Relax [8] application in Rosetta was used to remove these steric clashes. The command line of the Relax was:

relax.linuxgccrelease –database $PATH_TO_DB -in:file:fullatom -relax:quick -in:file:s start.pdb -use_input_sc -relax::constrain_relax_to_start_coords.

REFERENCES

1. Frishman D, Argos P. Knowledge-based protein secondary structure assignment. Proteins. 1995; 23:566–579.
2. Altschul SF, Gish W, Miller W, Myers EW and Lipman DJ. Basic local alignment search tool. J Mol Biol. 1990; 215:403–410.
3. Coutsias EA, Seok C, Wester MJ, Dill KA. Resultants and loop closure. International Journal of Quantum Chemistry. 2006; 106:176–189.
4. Mandell DJ, Coutsias EA, Kortemme T. Sub-angstrom accuracy in protein loop reconstruction by robotics-inspired conformational sampling. Nat Methods. 2009; 6:551–552.
5. Kaufmann KW, Lemmon GH, Deluca SL, Sheehan JH and Meiler J. Practically useful: what the Rosetta protein modeling suite can do for you. Biochemistry. 2010; 49:2987–2998.
6. Leaver-Fay A, Tyka M, Lewis SM, Lange OF, Thompson J, Jacak R, Kaufman K, Renfrew PD, Smith CA, Sheffler W, Davis IW, Cooper S, Treuille A, et al. ROSETTA3: an object-oriented software suite for the simulation and design of macromolecules. Methods Enzymol. 2011; 487:545–574.
7. Davis IW, Arendall WB, Richardson DC, Richardson JS. The backrub motion: how protein backbone shrugs when a sidechain dances. Structure. 2006; 14:265–274.
8. Conway P, Tyka MD, DiMaio F, Konerding DE, Baker D. Relaxation of backbone bond geometry improves protein energy landscape modeling. Protein Sci. 2014; 23:47–55.

Supplementary Table S1: Length range between α-carbons of different residue numbers[a]

Residue number	Length range (Å)
4	5.22–9.83
5	6.28–12.82
6	8.69–15.77
7	9.99–18.58
8	10.70–21.21

[a]Between the same residue numbers, the average length of C_α-C_α in a helix segment is usually shorter than that in a strand segment. The lower limit of length range is the average length value of α-helices and the upper limit is the average length value of β-strands.

Supplementary Table S2: Prediction results of five selected anchor residues predicted by KFC2, PredHS and Robetta

Residue	KFC2[a]				PredHS[b]		Robetta[c]	
	KFC2-A Conf	KFC2-A Class	KFC2-B Conf	KFC2-B Class	Ensemble score	Prediction result	$\Delta\Delta G_{bind}$	Prediction result
Y56	–0.26	–	0.18	Hotspot	–	–	4.05	Hotspot
R113	–0.12	–	–0.09	–	1.00	Hotspot	1.76	Hotspot
A121	0.58	Hotspot	–0.56	–	0.52	Hotspot	–	–
D122	1.22	Hotspot	–0.01	–	0.90	Hotspot	0.88	–
Y123	1.54	Hotspot	0.37	Hotspot	1.00	Hotspot	3.77	Hotspot

[a]KFC2 has two methods for hotspots prediction: KFC2-A and KFC2-B. A residue is predicted as the hotspot if the score of KFC2-A Conf or KFC2-B Conf is bigger than zero. [b]PredHS has two classifiers to predict hotspots: SVM and Ensemble. Here, we only give results predicted by the Ensemble classifier. [c]Robetta defines a residue as the hotspot when the $\Delta\Delta G_{bind}$ is bigger than 1 kcal/mol.

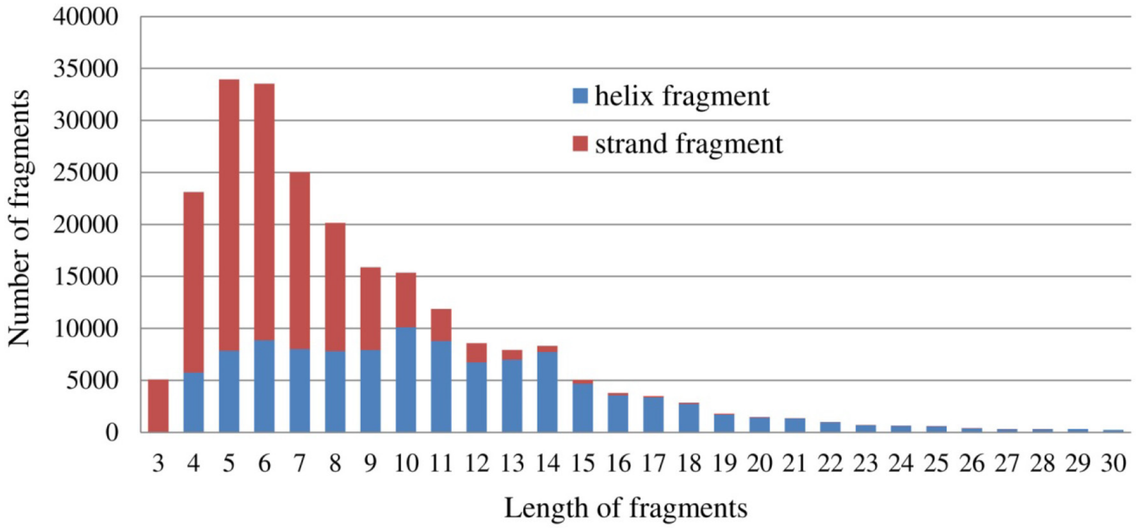

Supplementary Figure S1: Length distribution of scaffold fragments in the scaffold library. The shortest strand and helix fragment have 3 and 4 residues, respectively.

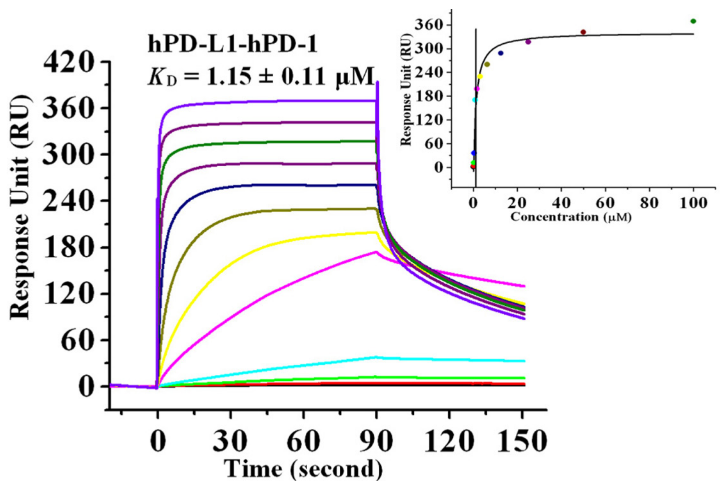

Supplementary Figure S2: SPR binding assay result of hPD-L1 binding to hPD-1. The K_D value is the mean of three independent experiments.

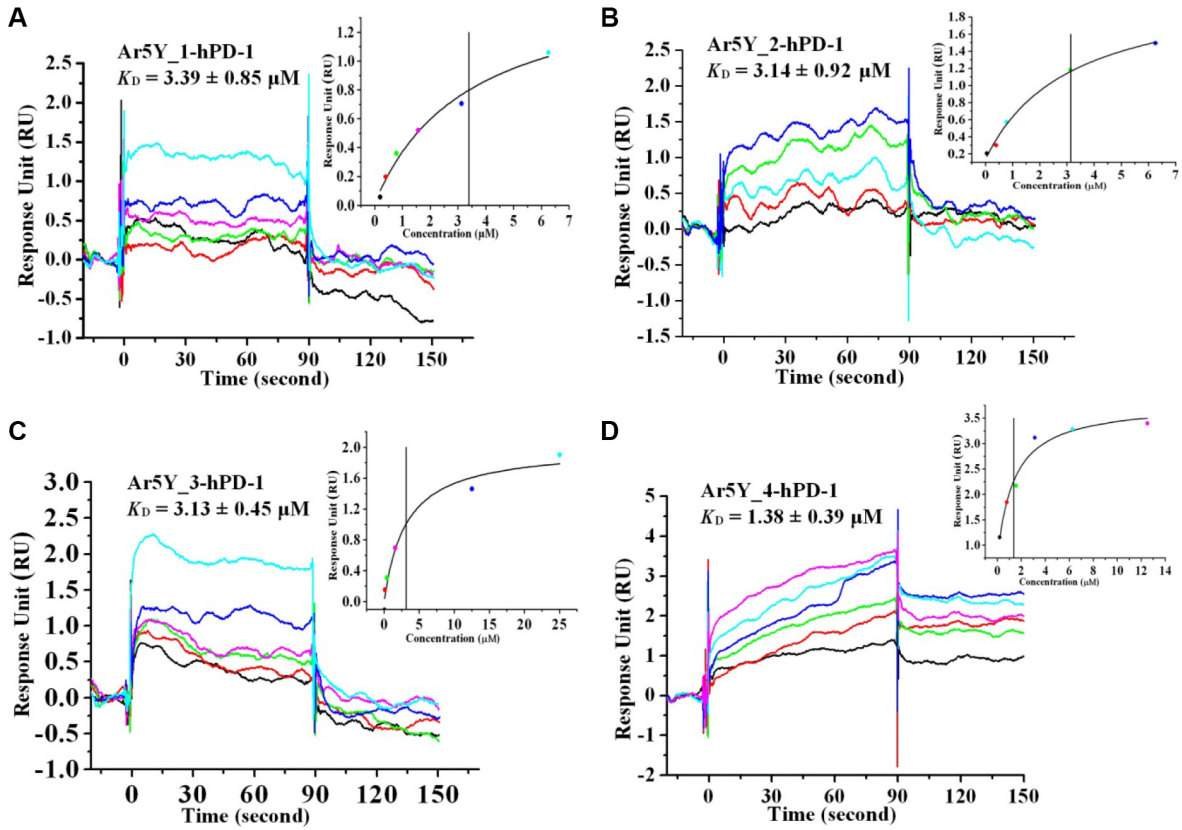

Supplementary Figure S3: SPR binding assay results of (A) Ar5Y_1, (B) Ar5Y_2, (C) Ar5Y_3 and (D) Ar5Y_4 binding to hPD-1. The K_D value is the mean of three independent experiments.

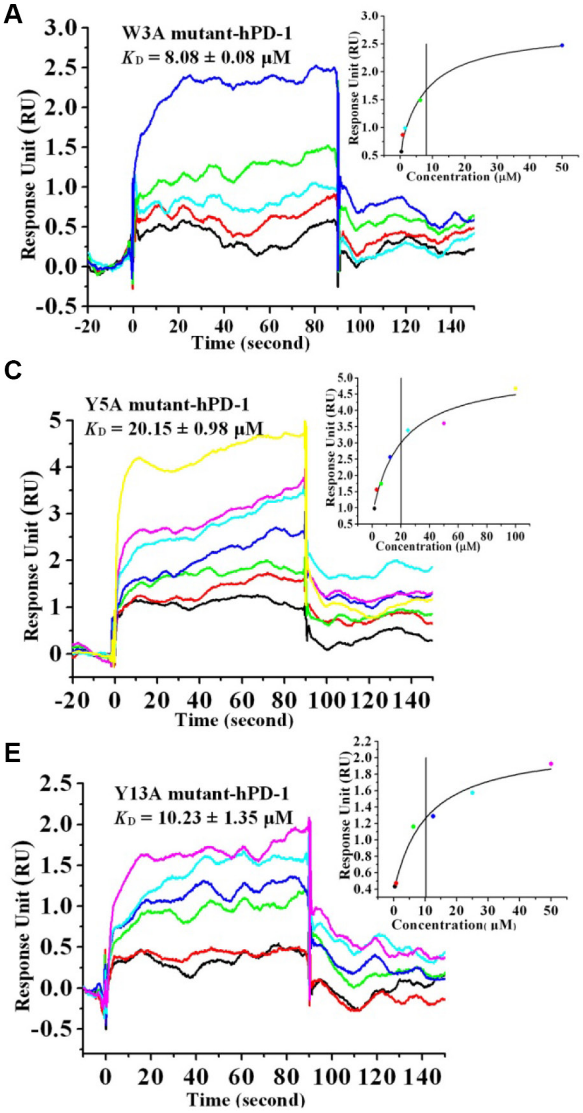

Supplementary Figure S4: SPR binding assay results of Ar5Y_4 mutants binding to hPD-1: (A) W3A mutant, (B) D4A mutant, (C) Y5A mutant, (D) R9A mutant and (E) Y13A mutant. The K_D value is the mean of three independent experiments.

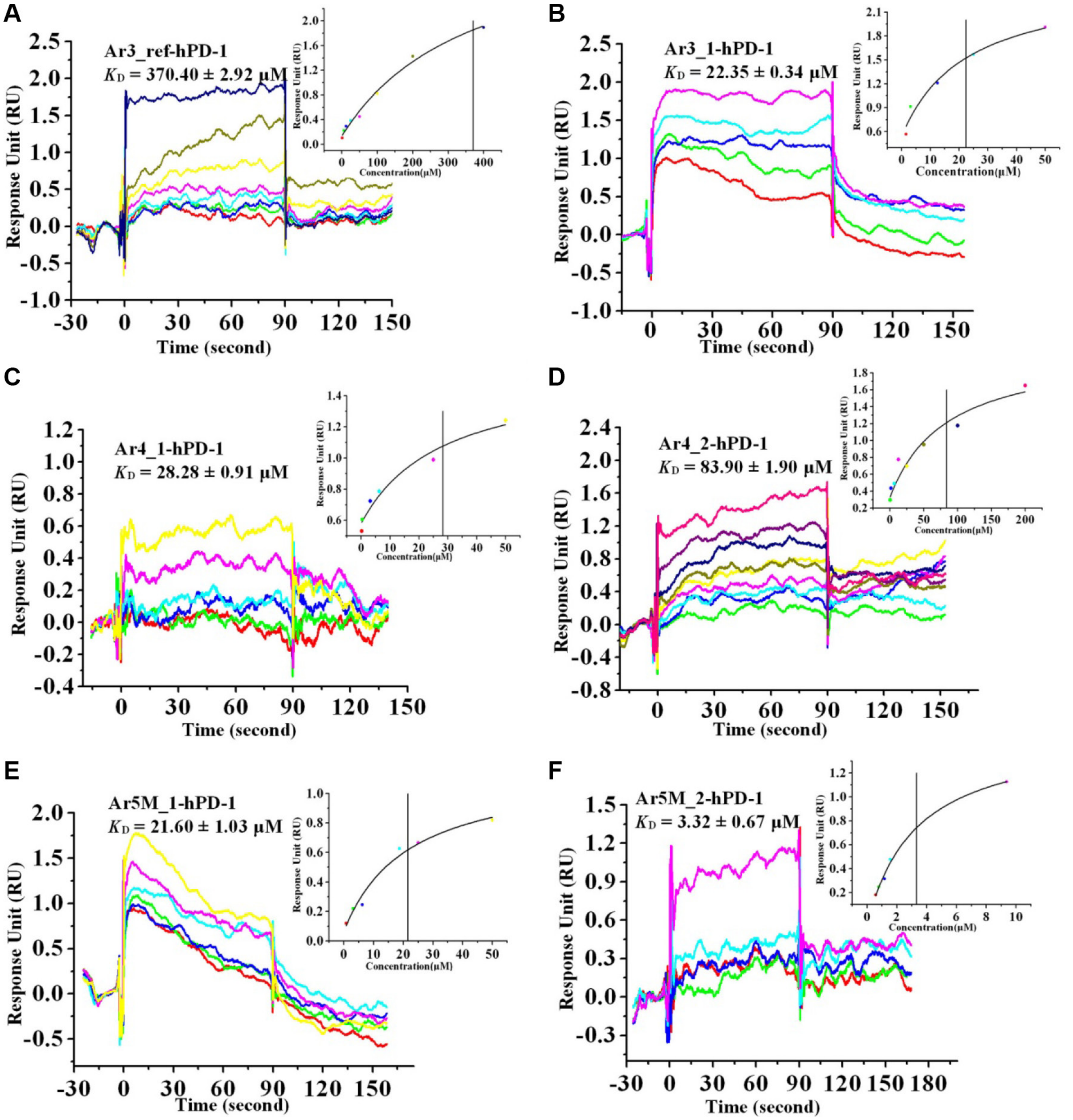

Supplementary Figure S5: SPR binding assay results of peptides designed with other anchor combinations binding to hPD-1: (A) Ar3_ref, (B) Ar3_1, (C) Ar4_1, (D) Ar4_2, (E) Ar5M_1 and (F) Ar5M_2. The K_D value is the mean of three independent experiments.

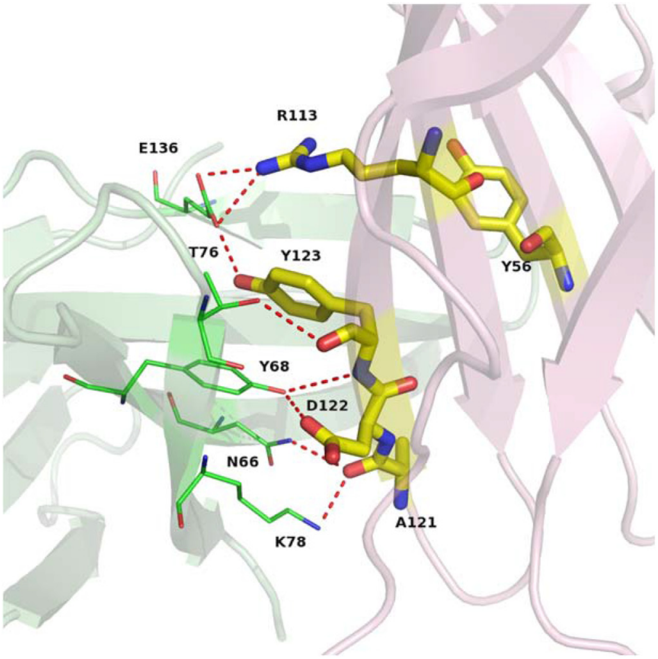

Supplementary Figure S6: Positions of five selected anchor residues at the hPD-1/hPD-L1 interface. hPD-1 and hPD-L1 are shown in palegreen and lightpink cartoon, respectively. The selected anchor residues Y56, R113, A121, D122 and Y123 are highlighted as yellow sticks, residues in hPD-1 that interact with the selected anchor residues are shown in green lines. Hydrogen bonds are depicted as red dashed lines.
